# Plate-associated localized osteitis in mini-pig by biofilm-forming Methicillin-resistant *Staphylococcus* *aureus* (MRSA): establishment of a novel experimental model

**DOI:** 10.1007/s00068-022-01894-2

**Published:** 2022-02-24

**Authors:** Carina Jaekel, Ceylan D. Windolf, Martin Sager, Lena M. Wollschläger, Martin Hoffmanns, Jan P. Grassmann

**Affiliations:** 1 Department of Orthopedics and Trauma Surgery, Medical Faculty, Heinrich Heine University Düsseldorf, Moorenstraße 5, 40225 Düsseldorf, Germany; 2 ZETT–Zentrale Einrichtung für Tierforschung und wissenschaftliche Tierschutzaufgaben, Heinrich Heine University Düsseldorf, Universitätsstraße 1, 40204 Düsseldorf, Germany; 3 Department of Diagnostic and Interventional Radiology, Medical Faculty, Heinrich-Heine-University Düsseldorf, Moorenstrasse 5, 40225 Düsseldorf, Germany

**Keywords:** Plate-associated osteitis, MRSA, *Staphylococcus aureus*, Mini-pig, Biofilm

## Abstract

**Purpose:**

The increasing number of implant-associated infections during trauma and orthopedic surgery caused by biofilm-forming *Staphylococcus aureus* in combination with an increasing resistance of conventional antibiotics requires new therapeutic strategies. One possibility could be testing for different therapeutic strategies with differently coated plates. Therefore, a clinically realistic model is required. The pig offers the best comparability to the human situation, thus it was chosen for this model. The present study characterizes a novel model of a standardized low-grade acute osteitis with bone defect in the femur in mini-pigs, which is stabilized by a titanium locking plate to enable further studies with various coatings.

**Methods:**

A bone defect was performed on the femur of 7 Aachen mini-pigs and infected with Methicillin-resistant *S. aureus* (MRSA ATCC 33592). The defect zone was stabilized with a titanium plate. After 14 days, a plate change, wound debridement and lavage were performed. Finally, after 42 days, the animals were lavaged and debrided again, followed by euthanasia. The fracture healing was evaluated radiologically and histologically.

**Results:**

A local osteitis with radiologically visible lysis of the bone could be established. The unchanged high Colony-forming Units (CFU) in lavage, the significant differences in Interleukin (IL)-6 in blood compared to lavage and the lack of increase in Alkaline Phosphates (ALP) in serum over the entire observation period show the constant local infection.

**Conclusion:**

The study shows the successful induction of local osteitis with lysis of the bone and the lack of enzymatic activity to mineralize the bone. Therefore, this standardized mini-pig model can be used in further clinical studies, to investigate various coated implants, bone healing, biofilm formation and immune response in implant-associated osteitis.

## Introduction

The increasing incidence of implant-associated infections induced by *Staphylococcus aureus* (SA) in combination with growing resistance to conventional antibiotics requires novel therapeutic strategies. The opportunistic SA can be found in about 30% of all humans in the nasopharynx, throat and intestinal tract [1]. In addition, SA is clinically the most important Staphylococcal species and known for a wide range of infections. Therefore, it is according to the International Consensus Conference of 2018 for Musculoskeletal Infections, not surprising that the main cause of complications in orthopedic surgery is infection with SA. In some regions, Methicillin-resistant SA (MRSA) is involved in over 50% of bone infection cases. Some studies show that the rate of recurrent or persistent infection after a two-stage revision is still 33% [2]. For all orthopedic subspecialties, the cost per patient with bone infection is between $61,000 and $150,000 [3]. SA’s ability to form a biofilm complicates the treatment of implant-associated infections. This biofilm protects the bacteria by preventing immune cells from entering, phagocytosis and ROS killing [2]. Moreover, the biofilm increases SA’s ability to acquire/disseminate plasmid-based antibiotic resistance determinants by horizontal gene transfer [4]. According to recent studies, SA migrates into a network of lacunae and canaliculi, being inaccessible to an immune response as well as an antibiotic therapy, thus surviving for several decades. This explains the high persistence of the disease. The harmful effects of SA in this network is leading to cell death of the osteocytes, which influences the entire bone metabolism and is resulting in chronic osteitis [2, 5]. Therefore, the highest priority is to prevent SA from adhering to implants and, above all, to form a biofilm.

There are numerous studies investigating implant coatings or antibiotic infused calcium sulfate/hydroxyapatite (CAS/HA) insets to prevent osteitis by SA. Silver coatings are the basis of many in vitro and in vivo studies [6–9]. However, Argawal showed that silver < 1 ppm is not able to reduce the infection rate, silver > 1.5 ppm, but can increase the cytotoxicity in the mammalian cell [10, 11]. Furthermore, gentamicin was enriched with the osteoinductive growth factor BMP2 in a rat model [12]. Also, in a rat model the implant was coated with gentamycin in a polylactide carrier matrix [13]. Li investigated vancomycin in a PEG–PLC matrix on rabbits and Diefenbeck used gentamycin coated implants on a plasma chemical oxidized titanium alloy pin in rats [14, 15]. In addition, numerous studies investigated antibiotic-enriched-hydroxyapatite coating or other surface modifications of orthopedic and dental [16–19]. Despite this large spectrum of studies, almost all were only conducted with Methicillin-sensible SA (MSSA) in small animal models. Besides the study by Stewart, who used a plate in sheep as an implant, and Li, who performed the studies with an MRSA in rabbits [20, 21].

In 2014, our working group was able to show that an implant coating with lysostaphin prevents MSSA osteomyelitis in a mouse model [22]. Unfortunately, due to the small size of the femur, it was not possible to change the plates of the mice in an existing infection. Lysostaphin destroys sessile bacteria in a biofilm and can also damage the extracellular biofilm matrix [23]. Moreover, the antibacterial potency of lysostaphin is well documented in animals and humans, even for infections with MRSA [24–26].

The immune system of mice is only 10% identical to the human immune system, whereas that of pigs is up to 80% identical. Furthermore, as pigs react similarly to infections (e.g., SA), they are very suitable for studies on infection and osteitis [27].

Therefore, the aim of this study was to establish a mini-pig model of a low-grade acute implant associated MRSA osteitis, simulating the clinical situation of a one-stage revision with plate replacement.

## Materials and methods

### Animals and ethics statement

For the induction of osteitis in the establishment group (control group without infection induction not relevant) seven 2-year-old Aachen mini-pigs (5 male, 2 female) with an average weight of 64 kg were used for the study (animal facility of the Heinrich-Heine University Düsseldorf; Zentrale Einrichtung für Tierforschung und wissenschaftliche Tierschutzaufgaben, ZETT, Germany). All animals were kept in separate stalls with an 12 h light/dark cycle. All animal procedures were carried out under local and national ethical guidelines and were approved by the regional ethical committee, Regional Office for Nature, Environment and Consumer Protection Nordrhein-Westfalen, Germany, with the ethical approval ID 84–02.04.2017.A181.

### Bacterial inoculum

The biofilm forming MRSA strain ATCC 33592 was cultivated in BactoTryptic Soy Broth overnight and afterwards diluted 1:10. The average inoculation CFU was 10^5^.

### Low-grade acute osteitis model

All operations were conducted in an aseptic operating room of our local animal facility. 4 weeks after acclimatization, the operations were performed. For premedication we used ketamine 10 mg/kg i.m., azaperone 5 mg/kg i.m., diazepam 10–20 mg i.m., atropine 0.5 mg i.m.. The anesthesia was performed via thiopental 5 mg/kg i.v. and 2% isoflurane/oxygen mixture for the induction of anesthesia. The anesthesia was maintained with 1.3% isoflurane/oxygen mixture, analgesia with buprenorphin 0.3 mg i.v.. The fascia was opened after skin incision in sterile. A standardized bone defect of 2.8 × 5 mm was created in the midshaft of the femur with an LCP twist drill (DePuy Synthes). A 5-hole LCP titanium plate 3.5 (DePuy Synthes) was then modeled onto the bone and infected with 5 × 5 µl with a total average CFU of 1 × 10^5^ (ATCC 33592 MRSA). After the bacteria dried, the plate was implanted laterally on the femur with four locking screws (Stardrive^®^, 3.5 mm, self-tapping, titanium, DePuy Synthes). The animals received oral analgesic treatment with meloxicam 0.4 mg/kg once a day and 3 × metamizol 20 mg/kg. buprenorphin 0.3 mg i.m. was given for the night. Systemic antibiotic therapy with enrofloxacin 2.5 mg/kg was performed from the first to the third postoperative day. On day 14, the 5-hole LCP titan plate was removed, a debridement and a lavage were performed and an uninfected 7-hole LCP titan plate with six locking screws (Stardrive^®^, 3.5 mm, self-tapping, titanium, DePuy Synthes) was implanted. A blood sample was collected. On day 42, the animals were sacrificed (thiopental overdose), a blood sample was gained, and a final sample from the lavage was collected from the surgical field. 

### Counts of colony-forming units (CFU)

The number of CFU was elevated in the lavage on days 14 and 42. Lavage were collected during debridement as described above. 200 µl of the lavage fluid were serially diluted in Phosphate-Buffered Saline (PBS). Four replicates were made of 10 µl of each dilution planted on Columbia agar plates with 5% sheep blood. The plates were incubated for 24 h at 37 °C. Thereafter, the colonies were counted. The results are expressed in CFU/ml lavage fluid (fourfold approach).

### Radiographic and histological analysis

The plate position and bone healing were controlled radiologically on days 0, 14 and 42 with a standard digital X-ray machine. After sacrificing the animals, the femora were harvested and fixed in formalin 4%. Bone fragments were generated at defined sites with an oscillating saw (Trauma Recon System, DePuy Synthes) and decalcified with a neutral EDTA solution for 8 weeks. 10 µm sections of the bone were stained with hematoxylin/eosin (HE).

### Analysis of local and systemic immune response

The local and systemic immune response was measured via polymorphonuclear leukocytes (PMN) as percentages of the total number of leucocytes in the lavage and blood samples using flow cytometry (FACSCalibur™; BD Pharmingen, Heidelberg, Germany) with an antibody (FITC Mouse Anti-Pig Monocyte/Granulocyte, BD Pharmingen). The samples were tested in duplicate.

### Quantification of IL-6 by ELISA

IL-6 levels in the lavage and blood were analyzed using a commercially available Swine IL-6 ELISA kit according to the manufacturer´s instructions (Thermo Scientific, Invitrogen, Waltham, USA) in a microplate reader (VICTOR X3 Plate Reader, PerkinElmer LAS, Rodgau, Germany). The samples were tested in duplicate. The lower detection limit for IL-6 was 45 pg/ml.

### Alkaline phosphatase level (ALP)

ALP activity, a non-specific marker for bone healing, was determined in serum. We used an AP Assay Kit measuring the Alkaline Phosphatase (AP) activity directly without pretreatment (Abnova, Taipei, Taiwan). This method utilizes p-nitrophenyl phosphate that is hydrolyzed by ALP into a yellow-colored product. The rate of the reaction is directly proportional to the enzyme activity and was measured at wavelengths of 405 nm 0 and 4 min after reaction (Victor X3, Plate Reader, PerkinElmer LAS, Rodgau, Germany). Samples were tested in duplicates.

### Statistical methods

All data are expressed as median and scatter dots. Data were tested for statistical significance with Mann–Whitney *U* test using GraphPad Prism5 (GraphPad Software, San Diego, CA): *p* values ≤ 0.05 were considered as significant.

## Results

### Clinical observations

**Fig. 1:**
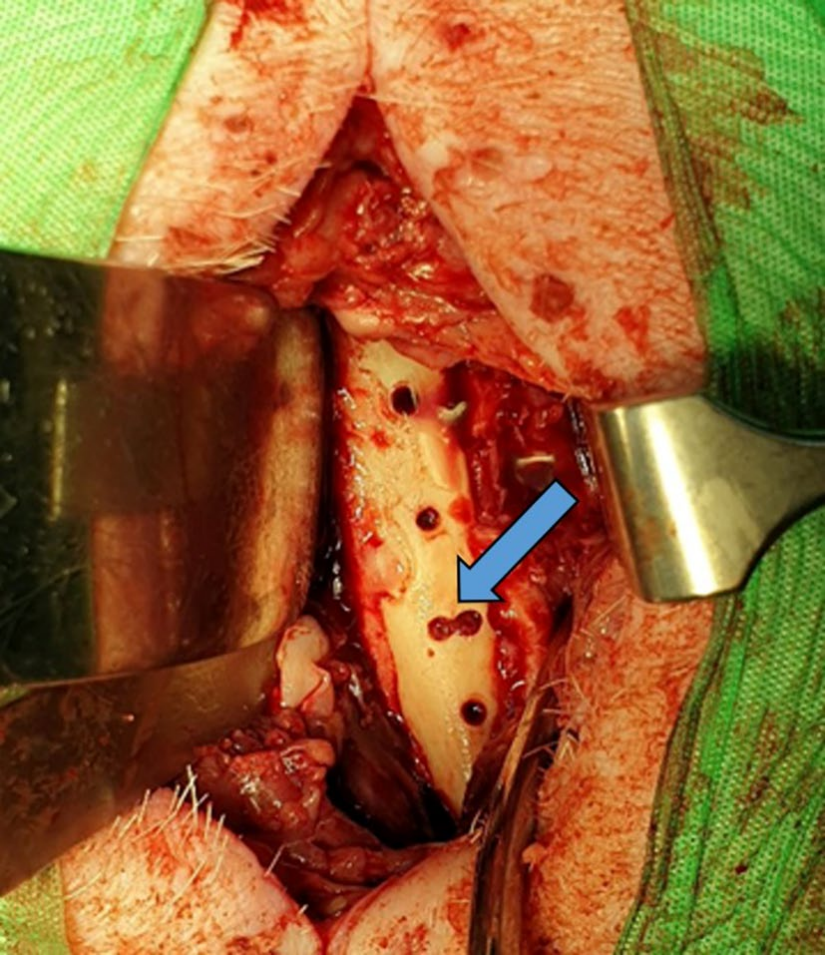
Bone defect. The blue arrow shows the extended defect zone on day 14 during plate change

All seven mini pigs tolerated the technique of the standardized bone defect of 2.8 × 5 mm (Fig. 1). They all returned to normal activity inside the cage and ate on their own. All animals survived throughout the study period without any plate breakage, fracture or other relevant adverse events and could be euthanized according to the study protocol.

### Low grade acute osteitis model

**Fig. 2:**
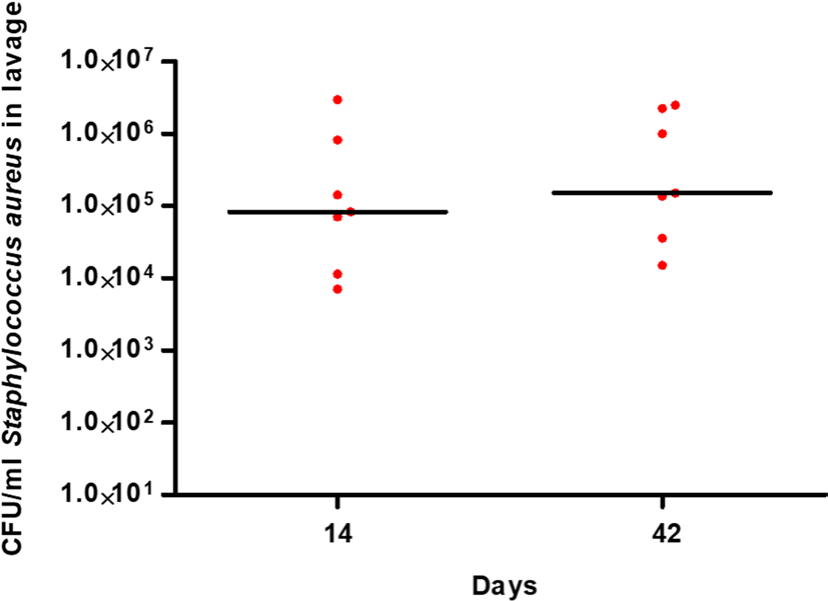
Bacterial load of *Staphylococcus aureus* in lavages. CFU levels in lavage remained consistently high over the entire observation period. *CFU*  colony-forming units

The bacterial load in the wound was determined by CFU on days 14 and 42 with an average inoculation CFU of 10^5^ (Fig. 2).

### Radiographic and histological analysis

**Fig. 3:**
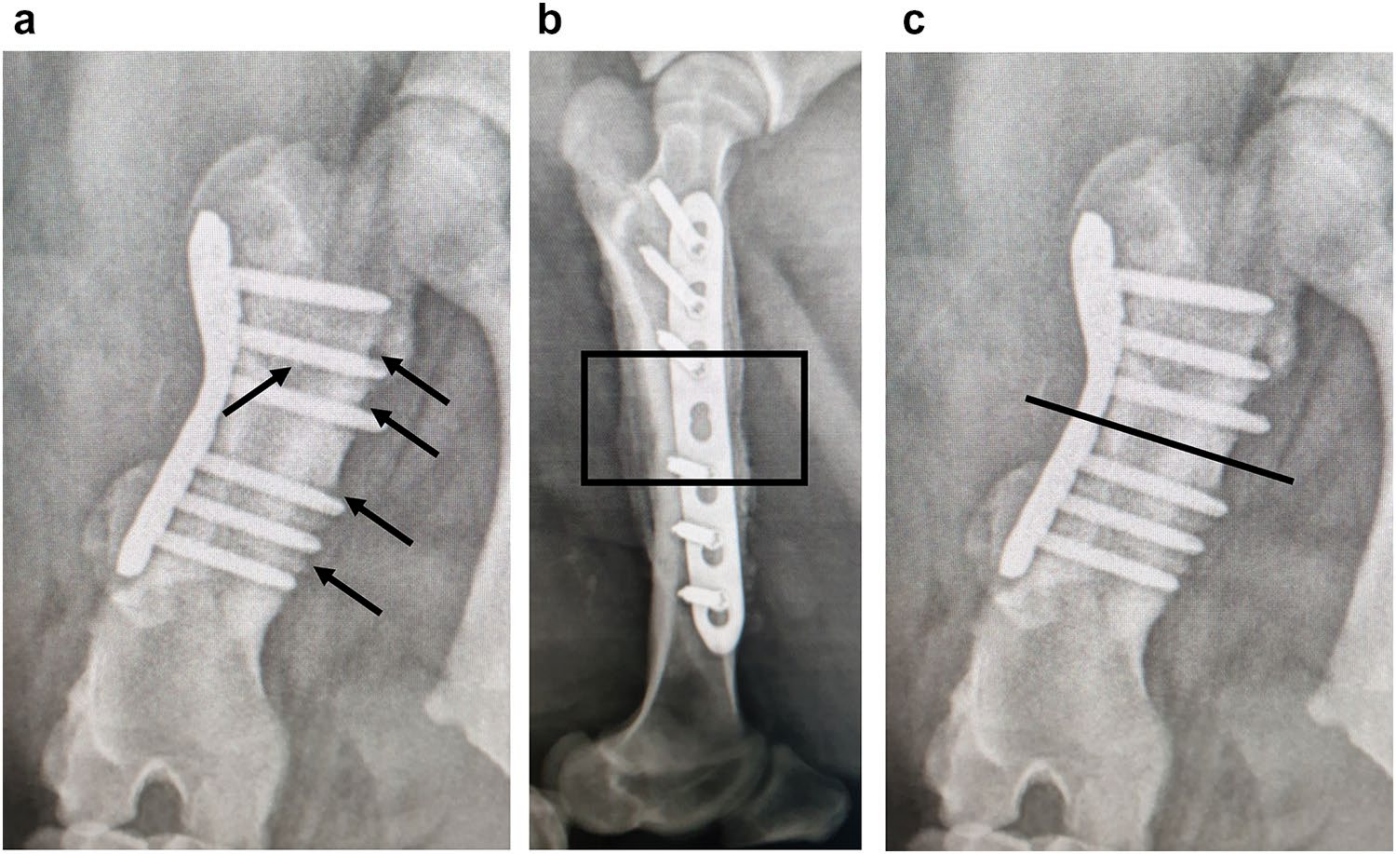
Radiographic lysis around the locking screws and periosteal reactions indicate osteitis on day 42. Arrows in **a** demonstrate the bone lysis around the screws on day 42. Box **b** and line **c** show the bone section for histology

**Fig. 4:**
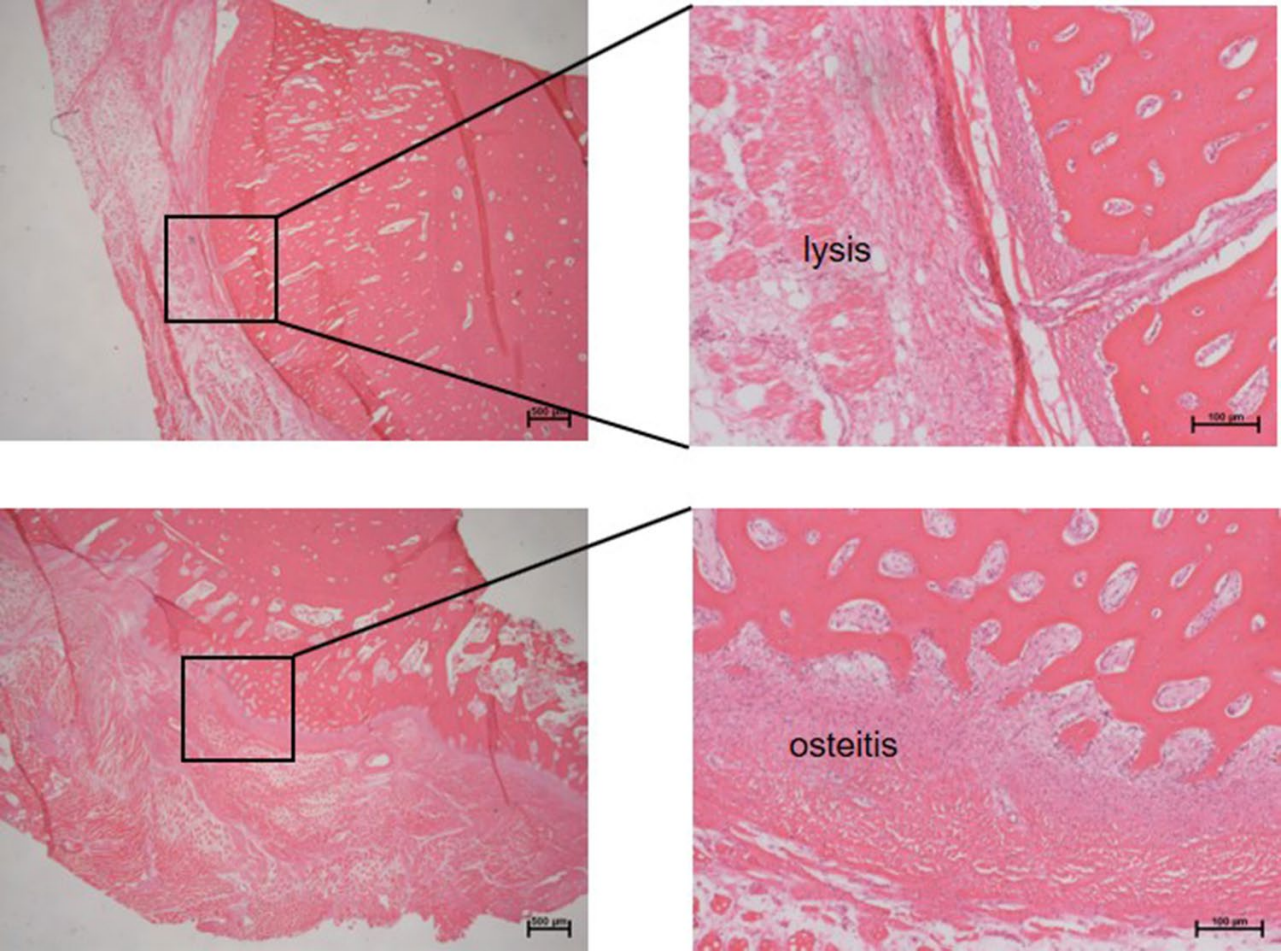
Histological representation of the bony changes in the presence of osteitis. Two different areas of the same bone section in the overview of periost and cortex (box left images) with the respective magnifications (right images)

Radiologically detectable lysis around the locking screws and periosteal reactions in all mini-pigs indicate osteitis on day 42. (Fig. 3a, b and c). Histologically illustrated osteitis and lysis of the bone (Fig. 4).

### Immune response

**Fig. 5:**
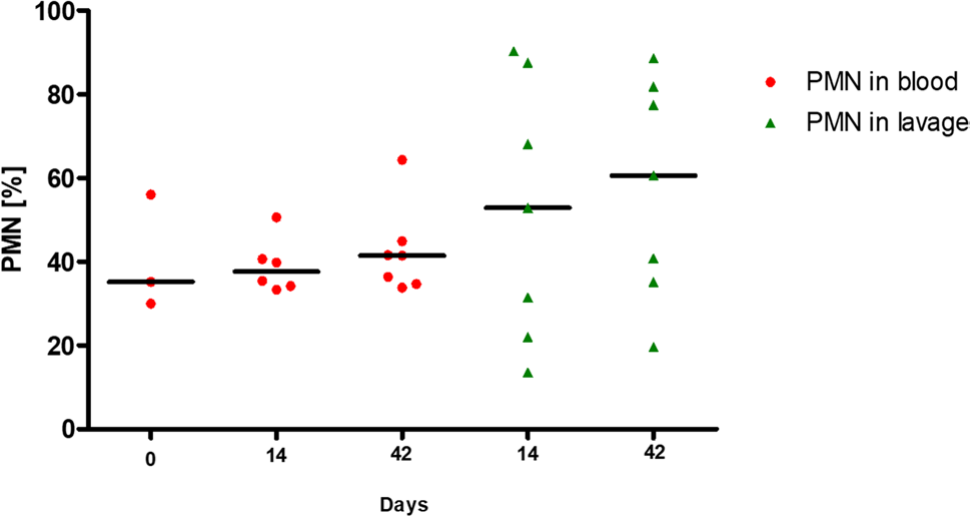
Neutrophil granulocytes in blood and lavage. There were no significant differences of PMN in blood compared to lavage on days 14 and 42. *PMN*: polymorphonuclear leukocytes

**Fig. 6:**
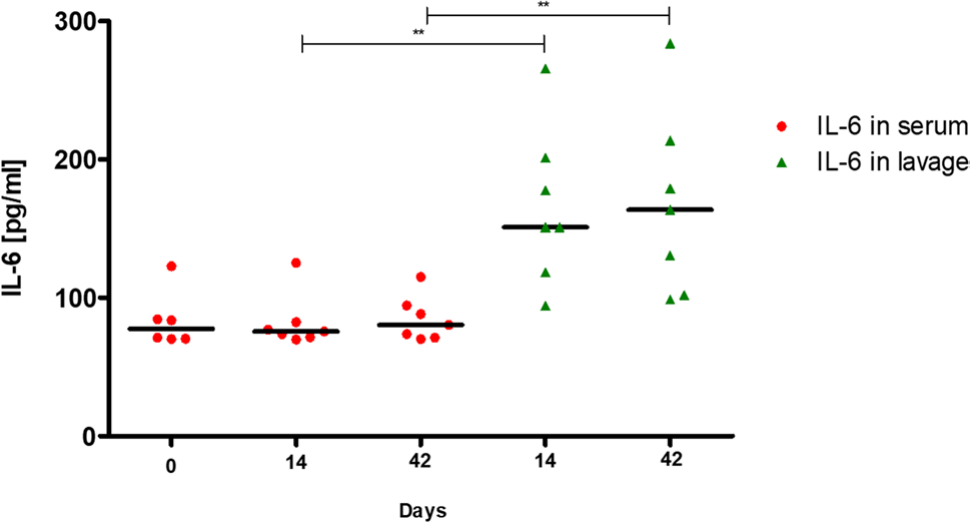
IL-6 in blood and lavage. IL-6 in lavage was significantly increased both on day 14 (*p* = 0.0023) and on day 42 (*p* = 0.0023) in comparison to IL-6 in blood. *IL-6:* Interleukin-6

Immune response was measured by detection of neutrophil granulocytes (Fig. 5) and IL-6 (Fig. 6) in lavages and serum.

**Fig. 7:**
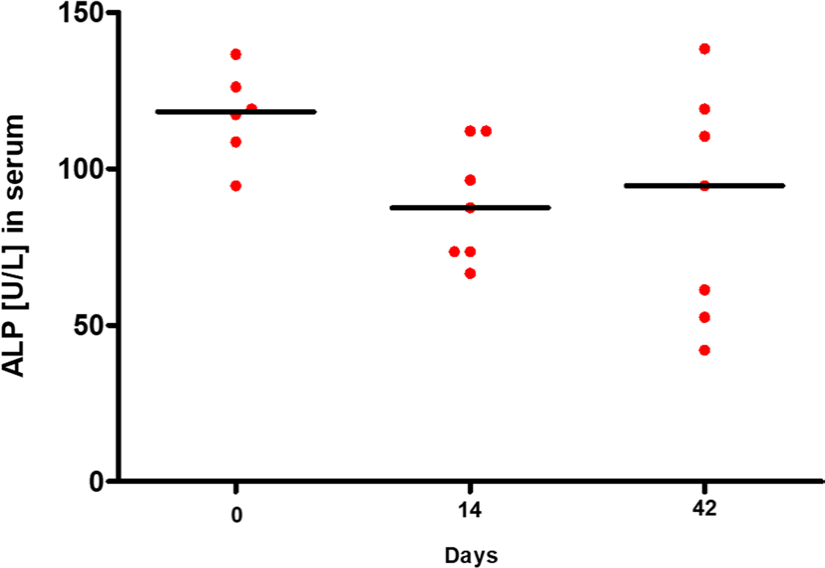
ALP in blood. ALP shows an overall very low level over the entire period, which does not change at any time. *ALP*: Alkaline Phosphatase

The consistently low ALP values show that no notable increased mineralization of the bone had occurred (Fig. 7). This can underpin the establishment of an osteitis.

## Discussion

Osteitis caused by an implant-associated infection remains one of the greatest challenges in musculoskeletal surgery. This study shows the successful induction of local osteitis with lysis of the bone and the lack of enzymatic activity to mineralize the bone. Therefore, this standardized mini-pig model can be used in further clinical studies.

As we already explain in the introduction, there are numerous studies that aim to prevent the colonization of SA in bone from the outset [28–32]. Despite this large spectrum of studies, almost all were only conducted with Methicillin-sensible SA (MSSA) in small animal models. In a standardized osteomyelitis mouse model, our research group was able to show that lysostaphin is a highly effective substance against SA [22, 33]. Lysostaphin, can penetrate biofilm and destroy the embedded bacteria even in a MRSA-caused infection [23, 34, 35].

The further established standardized osteitis mouse model has some limitations. On the one hand we could not perform a plate change in an existing infection, which would correspond to the clinical routine, due to the small size of the mice. On the other hand, pigs are 80% immunologically and constitutionally similar to humans and are, therefore, much more suitable for clinical models [27, 36]. Therefore, we have now established this osteitis model in mini-pigs to demonstrate the effectiveness of lysostaphin-coated plates after plate change in an established MRSA infection.

During the experiment, the mini-pigs were treated with systemic antibiotic therapy to avoid a systemic inflammation. Deliberately late we started an antibiotic therapy on the 1st post-op day, to give the bacteria time to develop a biofilm on the implant, which protects them there for systemic antibiotic therapy. The overall low local and systemic immune response with radiological and histological evidence of osteitis confirms the success of this standardized model so that we can conduct a study with lysostaphin-coated plates in the next step.

This study has some limitations. Due to model establishment no control (negative) group was performed. This approach was chosen for various reasons. The sole aim of this model establishment was to test, whether osteitis can be induced at all in such sensitive animals as pigs. Based on the model that has now been developed, further studies are already being planned, which compare osteitis and bone regeneration in infected as well as in non-infected mini-pigs with and without lysostaphin-coated plates.

Another limitation is the previous evidence of a local and systemic infection. Immunohistochemistral detection of biofilm formation on the orthopedic implants, the detection of bacteria by means of Giemsa staining in the histological sections as well as the CFU in biofilm on plates and the bony material additional to the CFU in lavage would be great approach. Furthermore, a more detailed analyzes of the bone healing by determination of bone-specific ALP (bs-ALP), procollagen peptide (PINP) and computertomography next to the X-ray would be helpful. We want to include these in future studies.

This study shows the successful induction of osteitis with lysis of the bone and the lack of enzymatic activity to mineralize the bone in mini-pigs. Therefore, this standardized mini-pig model can be used in further clinical studies, to investigate various coated implants, bone healing, biofilm formation and immune response in implant-associated osteitis.

## References

[1] Fitzgerald JR, Holden MTG (2016). Genomics of natural populations of *Staphylococcus*
*aureus*. Annu Rev Microbiol.

[2] Masters EA, Trombetta RP, Mesy Bentley KL, de, Boyce BF, Gill AL, Gill SR,  (2019). Evolving concepts in bone infection: Redefining “biofilm”, “acute vs. chronic osteomyelitis”, “the immune proteome” and “local antibiotic therapy”. Bone Res.

[3] Schwarz EM, Parvizi J, Gehrke T, Aiyer A, Battenberg A, Brown SA (2018). International Consensus meeting on musculoskeletal infection: research priorities from the general assembly questions. J Orthop Res.

[4] Savage VJ, Chopra I, O’Neill AJ (2013). *Staphylococcus*
*aureus* biofilms promote horizontal transfer of antibiotic resistance. Antimicrob Agents Chemother.

[5] de Mesy-Bentley KL, Trombetta R, Nishitani K, Bello-Irizarry SN, Ninomiya M, Zhang L (2017). Evidence of *Staphylococcus*
*aureus* deformation, proliferation, and migration in canaliculi of live cortical bone in murine models of osteomyelitis. J Bone Miner Res.

[6] Akiyama T, Miyamoto H, Yonekura Y, Tsukamoto M, Ando Y, Noda I (2013). Silver oxide-containing hydroxyapatite coating has in vivo antibacterial activity in the rat tibia. J Orthop Res.

[7] Brennan SA, Ni Fhoghlu C, Devitt BM, O’Mahony FJ, Brabazon D, Walsh A (2015). Silver nanoparticles and their orthopaedic applications. Bone Jt J.

[8] Gurunathan S, Han JW, Kwon D-N, Kim J-H (2014). Enhanced antibacterial and anti-biofilm activities of silver nanoparticles against Gram-negative and Gram-positive bacteria. Nanoscale Res Lett.

[9] Sheehan E, McKenna J, Mulhall KJ, Marks P, McCormack D (2004). Adhesion of *Staphylococcus* to orthopaedic metals, an in vivo study. J Orthop Res.

[10] Agarwal A, Weis TL, Schurr MJ, Faith NG, Czuprynski CJ, McAnulty JF (2010). Surfaces modified with nanometer-thick silver-impregnated polymeric films that kill bacteria but support growth of mammalian cells. Biomaterials.

[11] Pan C, Zhou Z, Yu X (2018). Coatings as the useful drug delivery system for the prevention of implant-related infections. J Orthop Surg Res.

[12] Min J, Choi KY, Dreaden EC, Padera RF, Braatz RD, Spector M (2016). Designer dual therapy nanolayered implant coatings eradicate biofilms and accelerate bone tissue repair. ACS Nano.

[13] Vester H, Wildemann B, Schmidmaier G, Stockle U, Lucke M (2010). Gentamycin delivered from a PDLLA coating of metallic implants: in vivo and in vitro characterisation for local prophylaxis of implant-related osteomyelitis. Injury.

[14] Li D, Lv P, Fan L, Huang Y, Yang F, Mei X (2017). The immobilization of antibiotic-loaded polymeric coatings on osteoarticular Ti implants for the prevention of bone infections. Biomater Sci.

[15] Diefenbeck M, Schrader C, Gras F, Muckley T, Schmidt J, Zankovych S (2016). Gentamicin coating of plasma chemical oxidized titanium alloy prevents implant-related osteomyelitis in rats. Biomaterials.

[16] Grenho L, Salgado CL, Fernandes MH, Monteiro FJ, Ferraz MP (2015). Antibacterial activity and biocompatibility of three-dimensional nanostructured porous granules of hydroxyapatite and zinc oxide nanoparticles–an in vitro and in vivo study. Nanotechnology.

[17] Yan L, Jiang D-M, Cao Z-D, Wu J, Wang X, Wang Z-L (2015). Treatment of *Staphylococcus*
*aureus*-induced chronic osteomyelitis with bone-like hydroxyapatite/poly amino acid loaded with rifapentine microspheres. Drug Des Dev Therapy.

[18] Oezel L, Buren C, Scholz AO, Windolf J, Windolf CD (2019). Effect of antibiotic infused calcium sulfate/hydroxyapatite (CAS/HA) insets on implant-associated osteitis in a femur fracture model in mice. PLoS ONE.

[19] Pearson JJ, Gerken N, Bae C, Lee K-B, Satsangi A, McBride S (2020). In vivo hydroxyapatite scaffold performance in infected bone defects. J Biomed Mater Res Part B.

[20] Stewart S, Barr S, Engiles J, Hickok NJ, Shapiro IM, Richardson DW (2012). Vancomycin-modified implant surface inhibits biofilm formation and supports bone-healing in an infected osteotomy model in sheep: a proof-of-concept study. J Bone Jt Surg.

[21] Li Y, Liu L, Wan P, Zhai Z, Mao Z, Ouyang Z (2016). Biodegradable Mg-Cu alloy implants with antibacterial activity for the treatment of osteomyelitis: in vitro and in vivo evaluations. Biomaterials.

[22] Windolf CD, Lögters T, Scholz M, Windolf J, Flohé S (2014). Lysostaphin-coated titan-implants preventing localized osteitis by Staphylococcus aureus in a mouse model. PLoS ONE.

[23] Walencka E, Sadowska B, Rozalska S, Hryniewicz W, Rózalska B (2005). Lysostaphin as a potential therapeutic agent for staphylococcal biofilm eradication. Pol J Microbiol.

[24] Kokai-Kun JF, Chanturiya T, Mond JJ (2009). Lysostaphin eradicates established *Staphylococcus*
*aureus* biofilms in jugular vein catheterized mice. J Antimicrob Chemother.

[25] Schindler CA, Schuhardt VT (1964). Lysostaphin: a new bacteriolytic agent for the *Staphylococcus*. Proc Natl Acad Sci USA.

[26] Wu J, Kusuma C, Mond J, Kokai-Kun J (2003). Lysostaphin disrupts *Staphylococcus*
*aureus* and *Staphylococcus* epidermidis biofilms on artificial surfaces. Antimicrob Agents Chemother.

[27] Meurens F, Summerfield A, Nauwynck H, Saif L, Gerdts V (2012). The pig: a model for human infectious diseases. Trends Microbiol.

[28] Boyle KK, Sosa B, Osagie L, Turajane K, Bostrom MPG, Yang X (2019). Vancomycin-laden calcium phosphate-calcium sulfate composite allows bone formation in a rat infection model. PLoS ONE.

[29] Stavrakis AI, Zhu S, Loftin AH, Weixian X, Niska J, Hegde V (2019). Controlled release of vancomycin and tigecycline from an orthopaedic implant coating prevents *Staphylococcus*
*aureus* infection in an open fracture animal model. BioMed Res Int.

[30] Xu C-P, Chen Y, Sun H-T, Cui Z, Yang Y-J, Huang L (2019). Efficacy of NEMO-binding domain peptide used to treat experimental osteomyelitis caused by methicillin-resistant *Staphylococcus*
*aureus*: an in-vivo study. Antimicrob Resist Infect Control.

[31] Zhang P, Qin J, Zhang B, Zheng Y, Yang L, Shen Y (2019). Gentamicin-loaded silk/nanosilver composite scaffolds for MRSA-induced chronic osteomyelitis. R Soc Open Sci.

[32] Zhang T, Wei Q, Zhou H, Zhou W, Fan D, Lin X (2020). Sustainable release of vancomycin from micro-arc oxidised 3D-printed porous Ti6Al4V for treating methicillin-resistant *Staphylococcus*
*aureus* bone infection and enhancing osteogenesis in a rabbit tibia osteomyelitis model. Biomater Sci.

[33] Windolf CD, Meng W, Lögters TT, MacKenzie CR, Windolf J, Flohé S (2013). Implant-associated localized osteitis in murine femur fracture by biofilm forming *Staphylococcus*
*aureus*: a novel experimental model. J Orthop Res.

[34] Geoghegan JA, Foster TJ (2015). Cell wall-anchored surface proteins of staphylococcus aureus: many proteins multiple functions. Current topics in microbiology and immunology.

[35] Gonzalez-Delgado LS, Walters-Morgan H, Salamaga B, Robertson AJ, Hounslow AM, Jagielska E (2020). Two-site recognition of *Staphylococcus*
*aureus* peptidoglycan by lysostaphin SH3b. Nat Chem Biol.

[36] Fairbairn L, Kapetanovic R, Sester DP, Hume DA (2011). The mononuclear phagocyte system of the pig as a model for understanding human innate immunity and disease. J Leukoc Biol.

